# Isolated collagenoma in an HIV-positive patient on ART: a case report

**DOI:** 10.1186/s12879-023-08548-7

**Published:** 2023-09-02

**Authors:** Kevin K. Wu, Masoud Movassaghi, Brittney DeClerck, Maggie Chow

**Affiliations:** 1 Keck School of Medicine, University of Southern California, Los Angeles, CA USA; 2 Department of Dermatology, University of Southern California, 1200 N. State Street, Room 3250, Los Angeles, CA 90089 USA

**Keywords:** HIV, ART, HAART, Lipoma, Lipodystrophy, Collagenoma

## Abstract

**Background:**

Collagenomas are rare connective tissue hamartomas composed of dermal collagen. Patients infected with human immunodeficiency virus (HIV) can present with HIV-related lipodystrophy or lipomas. There are no known associations between HIV and collagenomas.

**Case presentation:**

Here we describe a case of an isolated collagenoma in an HIV patient on ART. The lesion was a seven by four-centimeter subcutaneous nodule with no epidermal changes located on the occipital scalp. This lesion was excised, and histopathology showed thick and randomly arranged collagen bundles, consistent with a collagenoma.

**Conclusion:**

This case represents an isolated collagenoma presenting in a patient with HIV. It is unclear whether HIV or ART contributed to the development of this collagenoma. Treatment of collagenomas include surgical excision and intralesional corticosteroids. In addition to lipoma or lipodystrophy, it is important to keep collagenoma in the differential diagnosis in a patient presenting with an isolated large indurated subcutaneous nodule.

## Background

Connective tissue nevi are hamartomas located in the dermis and can consist of collagen, proteoglycans, or elastin [1]. Collagenomas are a type of connective tissue nevus characterized by an increased composition of dermal collagen. The pathophysiology of collagenomas involves increased Type I collagen production with decreased levels of collagenase.

Collagenomas can be subtyped into inherited and acquired forms [1]. Inherited forms of collagenoma include familial cutaneous collagenoma and shagreen patches of tuberous sclerosis. Familial cutaneous collagenoma is an inherited autosomal dominant disorder presenting with many scattered dermal nodules distributed symmetrically throughout the trunk and extremities. Extracutaneous manifestations of familial cutaneous collagenoma include cardiomyopathy or heart conduction defects, which can lead to congestive heart failure. These nodules typically present around the age of 15–17 years old. Shagreen patches of tuberous sclerosis are also an inherited form of a collagenoma, which present with thickened tumors or plaques on the skin.

Acquired forms of collagenoma include eruptive collagenomas and isolated collagenomas. Eruptive collagenomas present with nodules like those seen in familial cutaneous collagenoma but without family history or cardiac manifestations. Isolated collagenomas, such as that seen in our patient, presents with solitary indurated plaques or nodules. Isolated collagenomas can be associated with a family history of collagenomas or be acquired without a family history. Isolated cerebriform collagenomas on the palmar and plantar surfaces have been associated with Proteus syndrome, which presents at birth with asymmetric overgrowth of the limbs, digits, head, or trunk. There have also been cases of isolated plantar collagenomas with no known associated syndromes or other clinical findings [2]. There is one case report of collagenoma presenting with clusters of reddish brown papules and plaques in a multidermatomal distribution on the left back and right shoulder in an immunocompetent patient [3]. Isolated collagenomas are rare, and there are currently no known associations between human immunodeficiency virus (HIV) and the development of collagenomas.

## Case presentation

A 42-year-old Hispanic male with a ten-year history of HIV with CD4 count of 600 cells per microliter (45.2%) (CD4 nadir was 34 cells per microliter at the time of diagnosis ten years prior) and a negative viral load on long-term ART therapy presented to our tertiary care dermatologic surgery clinic with a chief complaint of an asymptomatic bump on his occipital scalp. He was diagnosed with HIV in 2013 and has no other medical conditions. His HIV has been well-controlled with ART since his initial diagnosis. This patient had been on emtricitabine/tenofovir disoproxil fumarate during the initial five years of his HIV diagnosis and was subsequently switched to bictegravir/emtricitabine/tenofovir alafenamide, which he had been on for the past five years. The patient was on no other medications. This lesion was first noticed eight years ago in 2015 and gradually grew. There were no other lesions elsewhere on his body.

Five years prior to presentation in our clinic in 2018, an outside dermatologist attempted tumescent liposuction on this lesion with the assumption that it was a lipoma. During the procedure, the dermatologist had difficulty breaking up the lesion and was unable to remove the entirety of the lesion. No pathological tests were completed on the partially removed lesion. Following liposuction, the lesion was initially flatter but gradually grew again until he presented to our clinic.

The patient does not smoke cigarettes nor use recreational drugs. He drinks four to six alcoholic drinks per week and works as a disc jockey at a local night club.

On the occipital scalp, the patient had an asymptomatic seven by four-centimeter skin-colored, subcutaneous, oval-shaped, indurated, rubbery, and slightly mobile nodule with a smooth, ill-defined edge and no overlying epidermal changes. There were no cutaneous signs of tuberous sclerosis on the patient such as angiofibromas, ash-leaf spots, or ungual fibromas. Our initial differential diagnoses included HIV-associated lipodystrophy and less likely lipoma. HIV-lipodystrophy can present similarly with a large subcutaneous rubbery indurated nodule in the posterior neck/upper back area in a phenomenon known as “buffalo hump” [4]. However, presentation of a lesion more superior on the occipital scalp is less typical of a buffalo hump.

Laboratory evaluation showed an unremarkable complete blood count with a hemoglobin of 14.6 g per deciliter, white blood cell count of 5,200 per microliter, and platelet count of 263,000 per microliter. Metabolic panel was within normal limits. The patient’s viral load at the time of HIV diagnosis is unknown.

**Fig. 1:**
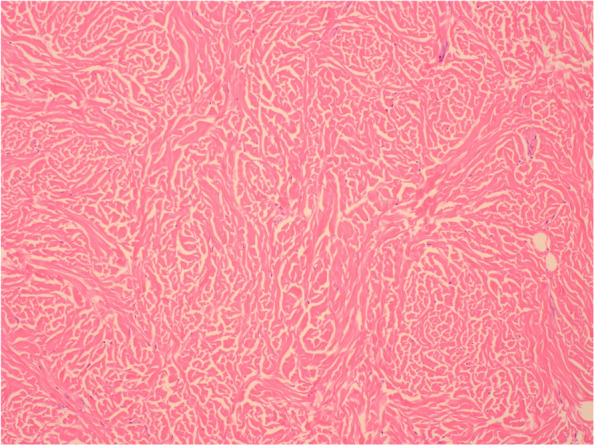
Hematoxylin & eosin staining of excision specimen at 100 × magnification

Our patient underwent surgical excision of the lesion in clinic under local anesthesia. During the procedure, tan-pink scar-like tissue was excised in pieces with no evidence of subcutaneous fat. Histopathology of this tissue showed thick and randomly arranged collagen bundles, consistent with an isolated collagenoma (Fig. 1). No histopathological stains were used.

## Discussion and conclusion

Here, we describe an isolated collagenoma in an HIV patient on long term ART. There has been one case in the literature describing eruptive collagenoma in an HIV patient [5]. However, causality in this case is questionable because eruptive collagenoma presented seven years prior to the diagnosis of HIV.

Histopathology of collagenomas shows thick and randomly arranged collagen bundles with diminished elastin and loss of normal empty spaces between bundles [6]. Special stains are not necessary to diagnose a collagenoma.

There are several differential diagnoses to consider when evaluating a painless subcutaneous, indurated nodule on the scalp in a patient of this age group, including lipoma, epidermal inclusion cyst, and pilar cyst. In a patient with HIV, it is important to consider HIV-associated lipodystrophy.

HIV has been classically associated with HIV-associated lipodystrophy and lipomas [4]. HIV infection can lead to increases in pro-inflammatory cytokines including TNF-alpha, IL-6, and IL-1beta. These cytokines can induce a stress response in adipocytes leading to lipodystrophy. Collagenomas, however, have not yet been reported in HIV patients. Collagen-producing fibroblasts are stimulated by IL-1, IL-10, and fibroblast growth factor. These cytokines are upregulated in HIV [7], which may play a role in the development of isolated collagenoma in our patient. More research is needed to determine the mechanism of the development of collagenoma in HIV patients.

ART medications can also cause lipodystrophy. Certain ART medications have a greater association with lipodystrophy in HIV patients, including protease inhibitors, zidovudine, didanosine, and stavudine [8]. The pathogenesis of lipodystrophy in HIV patients on ART is unclear but has been postulated to be due to metabolic abnormalities and inhibition of receptors involved in lipogenesis and lipolysis.

In addition to HIV-associated lipodystrophy and lipomas, HIV can also be associated with a host of other dermatologic conditions [9]. For example, due to impairment of the immune system, HIV patients have a greater risk of opportunistic skin infections, including deep fungal infections (e.g. cryptococcus, blastomycosis, histoplasmosis, and aspergillosis), viral infections (e.g. herpetic infections, molluscum contagiosum, and human papilloma viruses), and bacterial infections (e.g. cellulitis, syphilis, and bacillary angiomatosis). HIV is also associated with a higher risk of cutaneous malignancies such as Kaposi sarcoma, squamous cell carcinoma, basal cell carcinoma, and mycosis fungoides. Due to immune dysregulation, HIV patients are more likely to present with more severe inflammatory skin diseases as well, including psoriasis, seborrheic dermatitis, and atopic dermatitis.

Treatment options of collagenomas include surgical excision and intralesional corticosteroids [10]. There have been several reports of collagenomas treated by surgical excision resulting in complete resolution without recurrence. Cases of collagenomas treated with intralesional corticosteroids resulted in either substantial reduction in size or complete resolution [11, 12]. Attempts to treat collagenomas with cryotherapy have been largely unsuccessful [13].

We report an isolated collagenoma in an HIV patient. It is unclear if the pathophysiology of HIV or ART medications predispose patients to developing collagenomas. Collagenomas are rare, and further research is needed to elucidate the underlying mechanism of what might increase risk of collagenomas in HIV patients. It is important to keep collagenoma in the differential diagnosis when evaluating a patient with a large and indurated subcutaneous nodule.

## Data Availability

All data generated or analyzed during this study are included in this published article.

## Abbreviations

HIV: Human Immunodeficiency Virus
ART: Antiretroviral Therapy
